# Clinical Characteristics and Risk Factor Analysis for Lower-Extremity Amputations in Diabetic Patients With Foot Ulcer Complicated by Necrotizing Fasciitis

**DOI:** 10.1097/MD.0000000000001957

**Published:** 2015-11-06

**Authors:** I-Wen Chen, Hui-Mei Yang, Cheng-Hsun Chiu, Jiun-Ting Yeh, Chung-Huei Huang, Yu-Yao Huang

**Affiliations:** From the Division of Endocrinology and Metabolism, Department of Internal Medicine (I-WC, H-MY, C-HH, Y-YH), Molecular Infectious Disease Research Center, Division of Pediatric Infectious Diseases, Department of Pediatrics (C-HC); and Division of Trauma Plastic Surgery, Department of Plastic and Reconstructive Surgery, Chang Gung Memorial Hospital, Chang Gung University, Taoyuan City, Taiwan (J-TY).

## Abstract

Patients with diabetes are at a higher risk of having diabetic foot ulcers (DFUs) or necrotizing fasciitis (NF). The present study aims to examine the clinical characteristics and associated risk factors for lower-extremity amputation (LEA) in patients with DFU complicated by NF.

We retrospectively reviewed patients treated at a major diabetic foot center in Taiwan between 2009 and 2014. Of the 2265 cases 110 had lower-extremity NF. Limb preservation outcomes were classified as major LEA, minor LEA, or limb-preserved. Clinical characteristics, laboratory data, and bacterial culture results were collected for analysis.

Of the 110 patients with NF, 100 had concomitant DFUs (NF with DFU) and the remaining 10 had no DFU (NF without DFU). None of the NF patients without DFU died nor had their leg amputated. Two NF patients with DFU died of complications. The amputation rate in the surviving 98 NF patients with DFU was 72.4% (46.9% minor LEA and 25.5% major LEA). Seventy percent of the NF patients without DFU had monomicrobial infections (60% with *Streptococcus* species), and 81.4% NF patients with DFU had polymicrobial infections. Anaerobic organisms were identified in 66% of the NF patients with DFU. Multinomial logistic regression analysis revealed an association between high-grade Wagner wound classification (Wagner 4 and Wagner 5) and LEA (adjusted odds ratio [aOR] = 21.856, 95% confidence interval [95% CI] = 1.625–203.947, *P* = 0.02 and aOR = 20.094, 95% CI = 1.968–205.216, *P* = 0.01 for major and minor LEA, respectively) for NF patients with DFU. In addition, a lower serum albumin level was associated with major LEA (OR = 0.066, *P* = 0.002).

In summary, once DFUs were complicated by NF, the risk of amputation increased. Empirical treatment for NF patients with DFU should cover polymicrobial infections, including anaerobic organisms. The high-grade wound classification and low serum albumin level were associated with LEA.

## INTRODUCTION

Necrotizing fasciitis (NF) is a rare, rapidly progressive infection of subcutaneous tissue and fascia,^1^ characterized by widespread fascial necrosis. It is associated with a high rate of mortality, ranging from 6% to 76%,^2,3^ and 15% to 24% of patients with NF of the extremities require amputation.^3–5^ Early diagnosis, prompt surgery, and proper antibiotic usage are key factors to successfully treating NF.^6^ Diabetes mellitus is the major underlying disease in patients with NF, accounting for 15% to 71% of NF cases.^7^

Diabetic foot ulcer (DFU) is one of the major complications for patients with diabetes. An estimated 10% to 25% of diabetic patients will at any 1 time in their lifetime develop a DFU,^8–11^ which is the leading cause of nontraumatic lower-extremity amputation (LEA) worldwide.^10^ Unfortunately, studies on DFU complicated by NF are few. We found only 1 small-scale report from Singapore on 7 cases of NF among 202 patients with diabetic foot disease (a prevalence of 3.5%), 4 of whom (57.1%) required LEA.^9^

The aims of this study are to evaluate the clinical and microbiological features of patients with DFU complicated by NF and to analyze the risk factors for LEA.

## MATERIALS AND METHODS

### Study Subjects, Clinical Information, Diagnosis, and Management of NF

We retrospectively reviewed patients who were admitted to the Diabetic Foot Center for limb-threatening foot diseases at Chang Gung Memorial Hospital (a 3700-bed teaching hospital in Taiwan) between 2009 and 2014. This foot center is certified by the International Diabetes Federation-West Pacific Region. All patients were under the care of a multidisciplinary team composed of diabetologists, cardiologists, vascular surgeons, plastic surgeons and orthopedic surgeons, specialists in hyperbaric oxygen therapy, nutritionists, and nursing personnels.^12,13^ The Institutional Review Board of Chang Gung Memorial Hospital approved the study (No. 104-01535B).

Of the 2265 diabetic patients treated during the study period, 110 (4.9%) were identified as having lower-extremity NF, and all had type 2 diabetes mellitus. The diagnosis of NF was established at the time of surgery from the presence of necrotic fascial tissue, a lack of resistance of normally adherent muscular fascia to blunt dissection, the lack of bleeding of fascia during dissection, and the presence of foul smelling pus. The presence of asymmetric fascial thickening or gas^14^ in preoperative images (plain radiograph or computed tomography) was used as an adjunct to help make the diagnosis.^15,16^

Among the 110 patients diagnosed with lower-extremity NF, 100 had a concomitant foot ulcer (NF with DFU). The remaining 10 patients had no foot ulcer (NF without DFU) and were thus used as comparator group. There was no mortality for the NF patients without DFU. Two NF patients with DFU died of complications related to prolonged hospitalization: one of septic shock due to an intraabdominal infection and the other of pneumonia. These 2 cases were excluded from our analysis. In summary, the clinical and microbiological features of 108 NF patients were analyzed to identify the associated factors for LEAs.

The medical records included the patients’ age, gender, body mass index, duration of diabetes, concurrent cardiovascular disease, initial Wagner grading of the wound, and initial laboratory data (hemoglobin, white blood cell count, C-reactive protein, serum albumin, serum creatinine, glycated hemoglobin, total cholesterol, triglycerides, and low-density lipoprotein). The estimated glomerular filtration rate was calculated using the Modification of Diet in Renal Disease Study equation: 175 × serum creatinine (exp[−1.154]) × age (exp[0.203]) × (0.742 if female).^17^ DFUs were graded according to the Wagner wound classification system^18^ (grade 0: high-risk foot ulcer; grade 1: superficial ulcer; grade 2: deep ulcer penetrating to the tendon, bone, or joint; grade 3: deep ulcer with abscess or osteomyelitis; grade 4: gangrene in part of the forefoot; and grade 5: extensive gangrene of the foot). The wounds were graded by the diabetologist responsible and the first surgeon to see the patient.

### Microbiological Data

To ensure the quality of microbiological specimens, 67 out of 108 NF patients who had cultures taken in a sterilized operative room were studied. Among the NF patients without surgical specimen cultures, 40 had pus cultures collected from the open wound upon arrival to the hospital. One NF patient with DFU had no culture data available.

The organisms isolated from the surgical specimens were collected and transferred to standard aerobic and anaerobic swab/transport tubes. If a surgical specimen result was not available, pus culture specimens at the time of admission were obtained from the wound for culture. To avoid isolating colonizing flora, the wounds were thoroughly cleaned with normal saline before samples were obtained from the deeper pockets. Pus and exudates were collected from the margins and base of the ulcer, either with a syringe or with a sterile swab stick. The specimen was then transported in a clean and sterile transport system promptly to the laboratory.

Based on causative organism, patients with NF were categorized according to the most recent classification system:^6^ type I (70%–80% of cases, polymicrobial/synergistic), type II (20% of cases; usually monomicrobial), type III (gram-negative monomicrobial, including marine-related organisms), and type IV (fungal).

### Indications for Extremity Amputation

The criteria defined by the Global Lower Extremity Amputation Study were used by surgeons for surgical planning.^19^ A minor LEA was defined as any amputation distal to the ankle joint, while a major LEA was defined as any amputation through or proximal to the ankle joint. Those who did not require amputation were classified in the limb-preserved group.

The decision to perform an amputation was made by a team and performed by the plastic or orthopedic surgeons.^12^ The indications for LEA included failure of wound healing following proper treatments (antibiotic control, wound debridement, and revascularization) or uncontrolled infection or associated systemic comorbidities that impeded treatment.^12^

### Statistical Analysis

Demographic data and the clinical characteristics of the patients under study were represented as numbers with percentages for categorical variables and medians and interquartile ranges for continuous variables. The distribution of categorical data was compared between groups using Fisher exact test. Continuous data were compared between 2 groups (Table 1) using the Mann–Whitney *U* test, and among 3 groups (Table 4) using the Kruskal–Wallis test followed by Bonferroni post hoc multiple comparisons. To investigate the associated factors for LEA, multivariate multinomial logistic regression analysis was performed, in which the borderline significant (*P* < 0.10) variables in the bivariate analysis were introduced into the multivariate model. All data analyses were conducted using SPSS version 20 (IBM SPSS Inc., Chicago, IL).

**TABLE 1:**
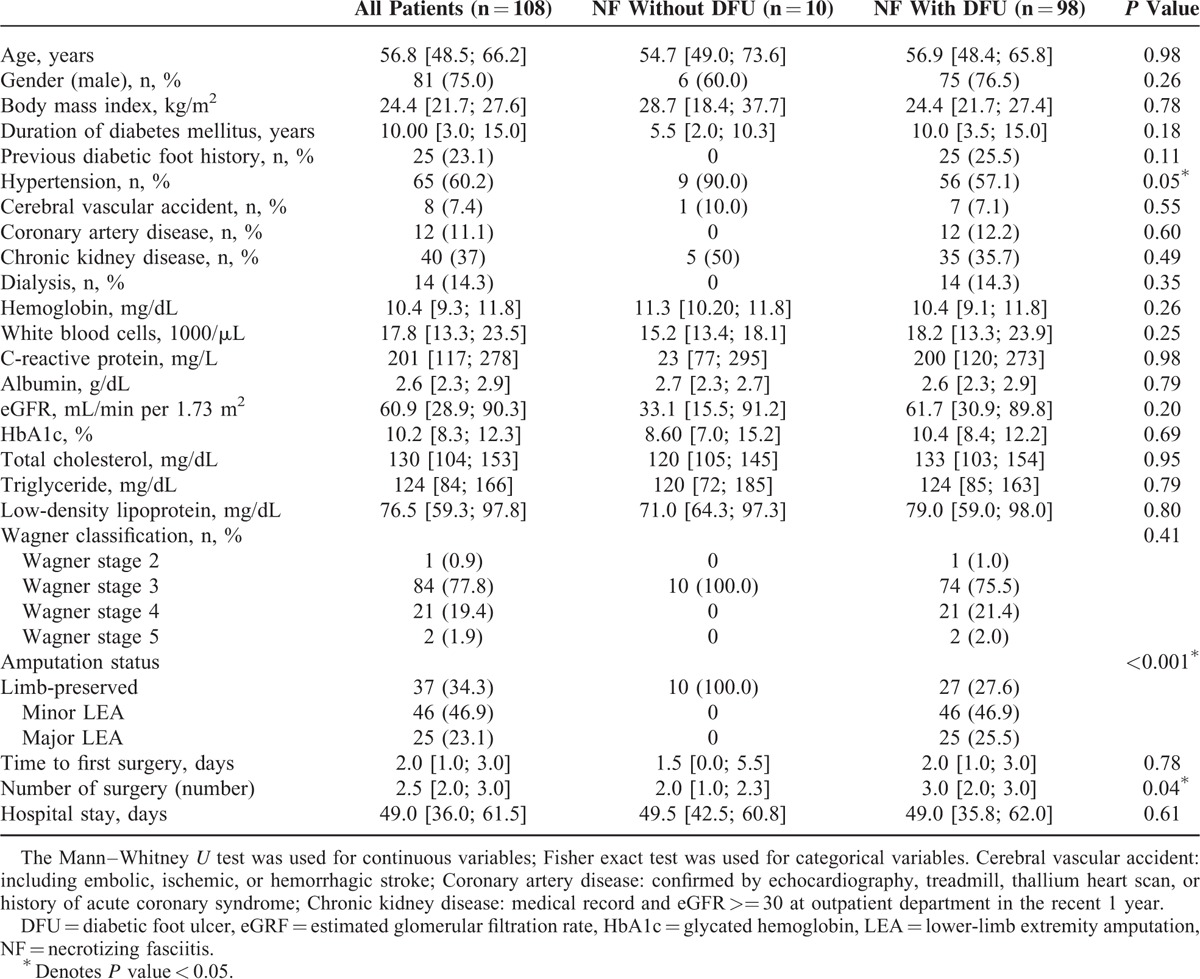
Demographics of the Diabetic Patients With Lower-Extremity Necrotizing Fasciitis Infections

## RESULTS

### Patient Characteristics

Of the 108 NF patients who survived, 81 (75.0%) were male with a median age of 56.8 years [48.5; 66.2]. The majority of the patients had poorly controlled diabetes with a median-glycated hemoglobin of 10.2% [8.3; 12.3]. The most common comorbidities were hypertension (60.2%), followed by chronic kidney disease (37%) and coronary artery disease (11.1%). Fourteen patients (14.3%) had end-stage renal disease-requiring hemodialysis (Table 1).

The patients received the first surgical procedure at a median period of 2.0 days [1.0; 3.0] after admission, and underwent a median of 2.5 surgical procedures [2.0; 3.0], such as fasciectomy, debridement, skin graft, or amputation. The median hospital stay was 49.0 days [36.0; 61.5] (Table 1).

### NF Patients With DFU Had Higher Rates of LEAs

None of the 10 NF patients without DFU had amputation. In contrast, NF patients with DFU had an amputation rate of 72.4% (minor LEA 46.9% and major LEA 25.5%). This rate is much higher than the overall amputation rate for diabetic patients with foot diseases.^20^

The NF patients with DFU tended to have had diabetes longer (Table 1), although the difference is not statistically significant. We note that 25.9% of the NF patients with DFU had a history of a previous foot ulcer. There were no differences in length of hospital stay between those with DFU and those without.

### Microbiological Findings

Table 2 lists the microorganisms isolated from the NF patients with and without DFU. One of the 10 NF patients without DFU and 6 of the 97 (6.2%) NF patients with DFU had no growth of any pathogen in their wound culture. Nine NF patients had bacteremia, all of whom had DFU. Seventy percent of the NF patients without DFU had monomicrobial infections, while 84.4% of the NF patients with DFU had polymicrobial infections (*P* < 0.001). Eighty percent of the NF patients without DFU had gram-positive bacteria, and *Streptococcus* sp. was the leading microorganism, accounting for 60.0% of the infections. For the NF patients with DFU, the rates of isolates of gram-positive bacteria, gram-negative bacteria, and anaerobes were 73.2%, 64.9%, and 66.0%, respectively. Compared with the NF patients without DFU, the presence of gram-negative (10%) or anerobes (20%) was significantly different (*P* = 0.001 or 0.007, respectively).

**TABLE 2:**
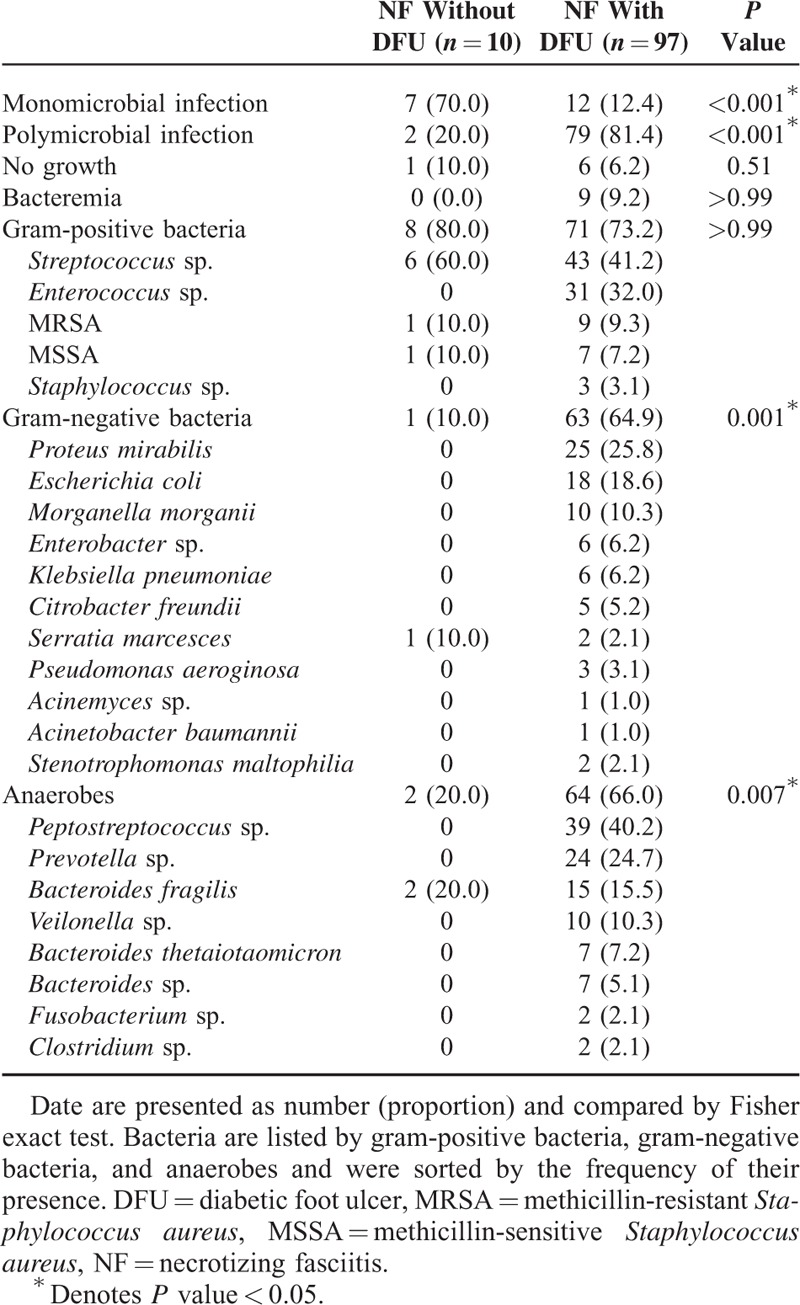
Microbiological Findings by Sites of Infection

According to the classification based on causative organisms,^6^ NF patients without DFU in this study were mostly classified as type-II NF (monomicrobial infections) and patients with DFU (polymicrobial infection) were categorized as type-I NF.

### Gas in Preoperative Plain Radiography as a Warning Sign for NF

**FIGURE 1:**
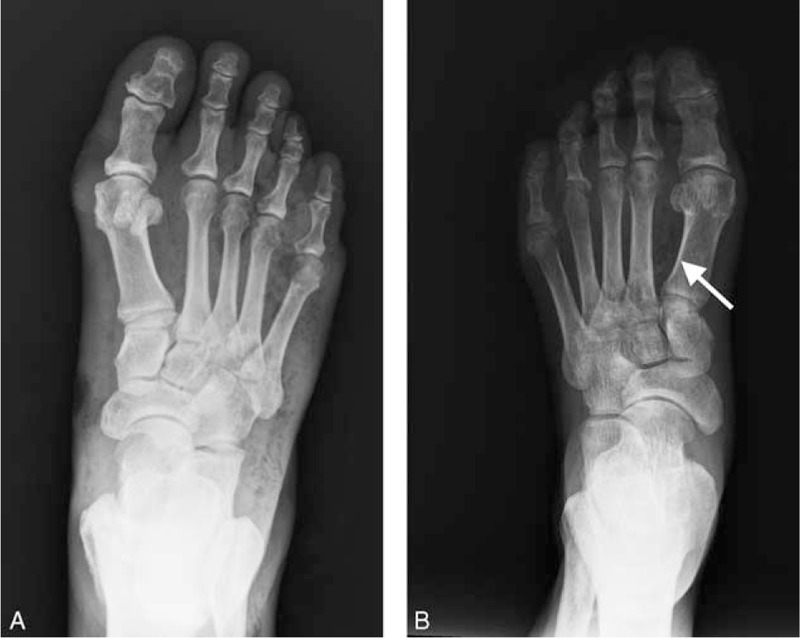
Gas formation revealed by plain radiograph in NF patients with DFU. (A) A 55-year-old male presented with 4th and 5th toes wet gangrene in the right foot. Subcutaneous emphysema was noted all over the foot. (B) A 50-year-old male presented with a grade 3 wound over plantar area of left foot. X-ray examination revealed air pockets between the big toe and 2nd toe (arrow). DFU = diabetic foot ulcer, NF = necrotizing fasciitis.

Gas in preoperative plain radiography was found in 76.1% of the NF patients with DFU (Fig. 1), and 42.9% of the NF patients without DFU. To study the correlation between tissue gas production and microbial infection, we analyzed the X-ray and microbiological findings in the NF patients with DFU (Table 3). Patients with polymicrobial infection (89.4%, *P* = 0.002) or anaerobic microorganisms (74.2%, *P* = 0.004) had a higher chance of gas detection in the radiographs.

**TABLE 3:**
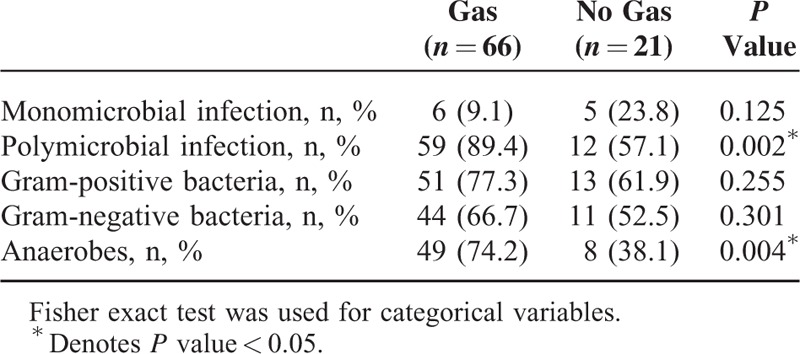
Microbiological Findings According to Presence of Gas in Plain Radiographs in the Patients Who Survived Necrotizing Fasciitis With Diabetic Foot Ulcer

### Risk Factor Analysis for Amputation in NF Patients With DFU

Result of post hoc comparisons showed that the serum albumin level was lower in the major LEA group (2.3 g/dL) than in the minor LEA group (2.6 g/dL, *P* = 0.002) and limb-preserved group (2.7 g/dL, *P* = 0.005) (Table 4). Only 1 patient (3.7%) in the limb-preserved group had a high-grade Wagner wound classification (Wagner stage 4 and 5) compared with 32.6% in the minor LEA group and 28% in the major LEA group (*P* = 0.04).

**TABLE 4:**
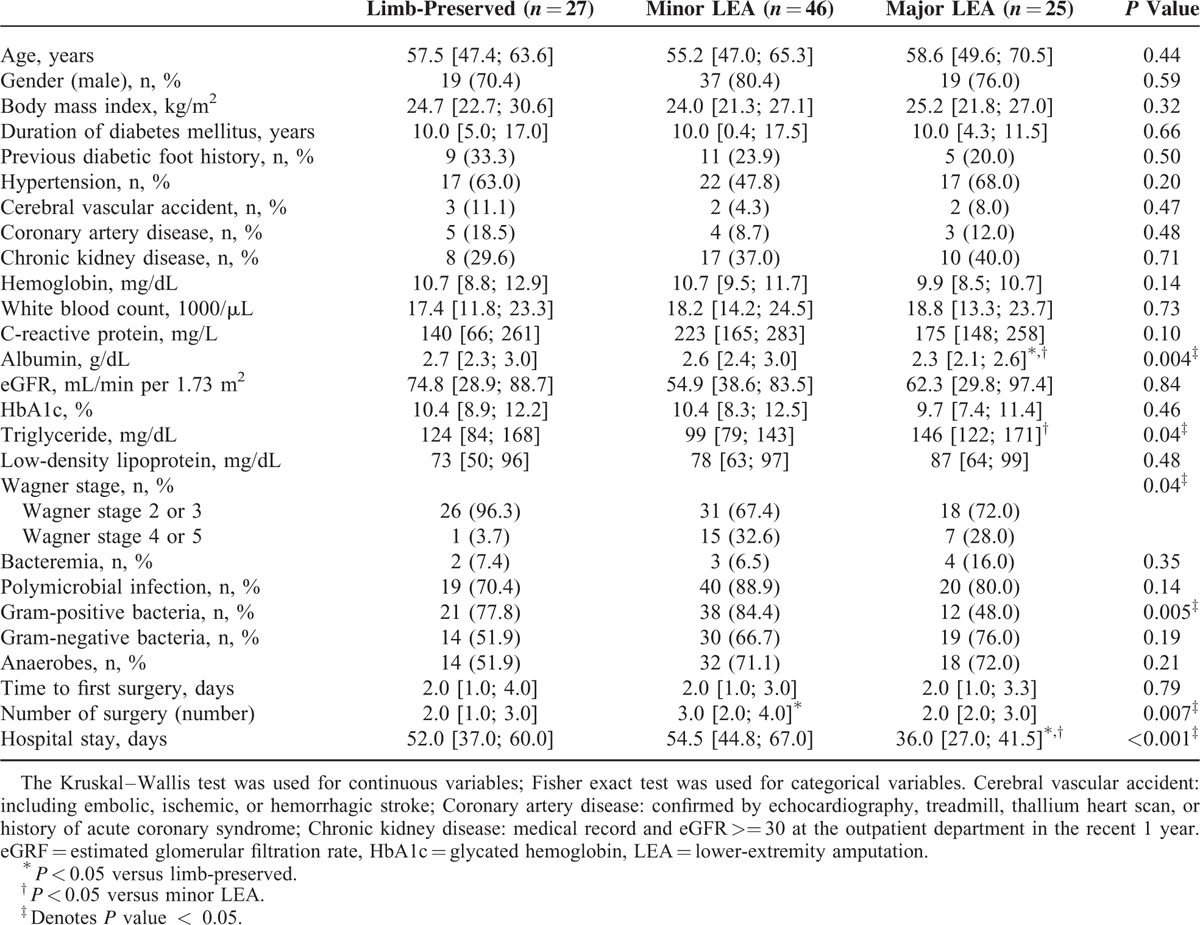
Demographic and Clinical Characteristics According to Extent of Lower-Extremity Amputation in the Patients Who Survived Necrotizing Fasciitis With Diabetic Foot Ulcer

Polymicrobial infection or the presence of bacteremia has no correlation with the need for amputation. Gram-positive bacteria were found more often in the minor LEA and limb-preserved groups compared with the major LEA group (*P* = 0.005) (Table 4).

Table 5 shows the multivariate multinomial logistic regression analysis adjusted for variables with *P* values less than 0.10 in the bivariate analysis. The results indicated that high-grade Wagner classification was associated with a higher risk of minor LEA (adjusted odds ratio [aOR] = 20.094, 95% confidence interval [95% CI] = 1.968–205.216, *P* = 0.01) and major LEA (aOR = 21.856, 95% CI = 1.625–203.947, *P* = 0.02). In addition, an increased C-reactive protein level was associated with a higher risk of minor LEA (aOR = 1.007, 95% CI = 1.001–1.013, *P* = 0.01). An increased albumin level was associated with a decreased risk of major LEA (aOR = 0.066, 95% CI = 0.011–0.409, *P* = 0.002). For every decrease of 1 mg/dL serum albumin, the risk of major LEA increases 15.2-fold.

**TABLE 5:**
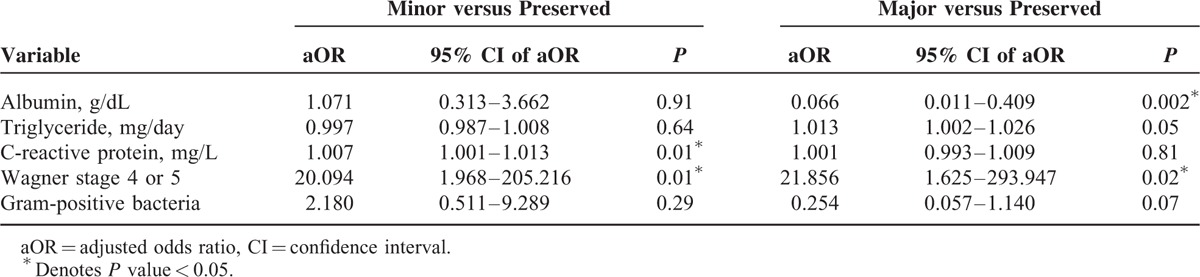
Factors Associated With Minor or Major Lower-Extremity Amputation by Multivariate Multinomial Logistic Regression Analysis

## DISCUSSION

The results of this study suggest that lower-extremity NF in diabetic patients is not rare, accounting for 4.9% of the cases in our diabetic foot center. A similar prevalence rate was reported in a small-scale study (3.5%, or 7 of the 202 patients with diabetic foot diseases).^9^ We note that most of our NF patients (90.9%) had a concomitant foot ulcer, and the amputation rate for them was still high at 72.4%. Therefore, when treating patients with a DFU, physicians should be alert to the concomitant presence of NF, as this complication will have a higher risk of LEAs.

NF patients without DFU in this study were mostly classified as type-II NF (monomicrobial infections), which is similar to the microorganisms reported from lower-extremity NF.^5^ Patients with DFU (polymicrobial infection) were categorized as type-I NF. Chronic and deep DFU may involve a more complex assortment of bacteria.^21^ Furthermore, the presence of anaerobic organisms in NF patients with DFU usually indicates a more extensive inflammation or necrosis.^21^ It is possible that these complex microbial infections in the NF patients with DFU originated from penetration of a pre-existing open DFU. We note that two thirds of the NF patients with DFU had aerobic and anaerobic bacterial coinfections. This suggests the importance of anaerobic cultures and the use of broad-spectrum or combination therapy to provide the best empirical antimicrobial coverage for these patients.

The crepitation noted in palpation and the presence of gas in plain X-ray images are usually signs for NF.^7^ In the NF patients with DFU, 76.1% of foot X-ray films were positive for gas presence. This is high compared with 4.9% to 57.4% for NF of other sites.^3^ The presence of polymicrobial infection or anaerobes may explain the higher rate of gas detection in the plain radiographs of these patients.

Several studies have reported that the Wagner wound classification type is a risk factor associated with amputation in patients with DFUs.^12,22^ In our NF patients with DFU, the foot ulcer grade is an associated factor for LEAs. Serum albumin level is an indicator of nutritional status^23^ and also a marker of inflammation.^24,25^ A lower serum albumin level has been reported to be associated with increased mortality in patients with NF.^26,27^ It is also known that nutrition plays a critical role in wound healing.^28^ A lower serum albumin level has been shown to be associated with major LEA in diabetic patients treated for foot ulcer.^12,29^ The addition of oral nutritional supplements to standard care has been shown to improve wound healing in DFU patients with a low albumin level, but not for patients with a normal albumin level.^30^ Further studies are needed to determine whether nutritional supplements benefit NF patients with DFU.

This study is limited by its retrospective design. Certain clinical features such as diabetic retinopathy were not available for analysis. Furthermore, patients at our referral foot center usually have limb-threatening foot ulcer. Therefore, patients with a lower grade of DFU were not enrolled in this study. The small size of NF patients without DFU limits the utility of statistical analysis.

In conclusion, early diagnosis and multidisciplinary treatment are essential when treating diabetic patients with lower-extremity NF. This study shows that NF is a complication for patients with DFU because it results in higher rates of LEAs. The presence of gas in plain X-ray images should alert clinicians to NF for diabetic patients with foot ulcer. Empirical treatment for NF patients with DFU should cover polymicrobial infections, including anaerobic organisms. A lower serum albumin level and high-grade Wagner wound classification are associated factors for LEA.
